# Effects of magnolol on UVB-induced skin cancer development in mice and its possible mechanism of action

**DOI:** 10.1186/1471-2407-11-456

**Published:** 2011-10-20

**Authors:** Chandeshwari Chilampalli, Ruth Guillermo, Xiaoying Zhang, Radhey S Kaushik, Alan Young, David Zeman, Michael B Hildreth, Hesham Fahmy, Chandradhar Dwivedi

**Affiliations:** 1 Department of Pharmaceutical Sciences, South Dakota State University, Brookings, SD 57007, USA; 2 ACEA Bio Ltd., Hangzhou, P.R. China; 3 Department of Biology and Microbiology, South Dakota State University, Brookings, SD 57007, USA; 4 Department of Veterinary and Biomedical Sciences, South Dakota State University, Brookings, SD 57007, USA

## Abstract

**Background:**

Magnolol, a plant lignan isolated from the bark and seed cones of *Magnolia officinalis*, has been shown to have chemopreventive effects on chemically-induced skin cancer development. The objectives of this investigation are to study the anticarcinogenic effects of magnolol on UVB-induced skin tumor development in SKH-1 mice, a model relevant to humans, and determine the possible role of apoptosis and cell cycle arrest involved in the skin tumor development.

**Methods:**

UVB-induced skin carcinogenesis model in SKH-1 mice was used for determining the preventive effects of magnolol on skin cancer development. Western blottings and flow cytometric analysis were used to study the effects of magnolol on apoptosis and cell cycle.

**Results:**

Magnolol pretreated groups (30, 60 μ g) before UVB treatments (30 mJ/cm^2^, 5 days/week) resulted in 27-55% reduction in tumor multiplicity as compared to control group in SKH-1 mice. Magnolol pretreatment increased the cleavage of caspase-8 and poly-(-ADP-ribose) polymerase (PARP), increased the expression of p21, a cell cycle inhibitor, and decreased the expression of proteins involved in the G2/M phase of cell cycle in skin samples from SKH-1 mice.

Treatment of A431 cells with magnolol decreased cell viability and cell proliferation in a concentration dependent manner. Magnolol induced G2/M phase cell cycle arrest in A431 cells at 12 h with a decreased expression of cell cycle proteins such as cyclin B1, cyclin A, CDK4, Cdc2 and simultaneous increase in the expression of Cip/p21, a cyclin-dependent kinase inhibitor. Magnolol induced apoptosis *in vivo *and *in vitro *with an increased cleavage of caspase-8 and PARP. Phospho-signal transducers and activators of transcription 3 (Tyr^705^), B-Raf, p-MEK, and p-AKT were down-regulated, whereas phosphorylation of ERK was induced by magnolol in A431 cells.

**Conclusions:**

Magnolol pretreatments prevent UVB-induced skin cancer development by enhancing apoptosis, causing cell cycle arrest at G2/M phase, and affecting various signaling pathways. Magnolol could be a potentially safe and potent anticarcinogenic agent against skin cancer.

## Background

In the United States, human, non-melanoma skin cancers are most frequently diagnosed in Caucasians, accounting for over 3.5 million cases each year [1]. American Cancer Society estimates indicated 11,980 deaths from skin cancer in 2011 [2]. The major causative factor for skin cancer is UV radiation from sunlight [3,4]. Both experimental and epidemiological evidences suggest UVB is an important component of solar radiation that acts as a complete carcinogen by initiating and promoting skin cancer [5,6]. Estimates show that one among five Americans will develop skin cancer [7]. UV radiation, besides resulting in characteristic DNA damage, also causes tumor promotion by inducing various signal transduction pathways which can lead to distinct cellular responses including cell proliferation, transformation, and cell death [8,9]. Mechanisms that suppress tumorigenesis involve the modulation of signal transduction pathways leading to arrest in the cell cycle progression or induction of apoptosis. It has been proposed that sunscreens alone are not sufficient in preventing skin cancer, thus there is a need for more effective ways to prevent this malignancy [10,11]. For this reason, chemoprevention of skin cancer by natural compounds has gained importance in recent years [12,13]. More than 1000 phytochemicals have shown chemopreventive effects against cancer [14-16], and one such phytochemical is magnolol, whose effects are investigated for the prevention of skin cancer in this study.

Magnolol and honokiol are phenolic compounds obtained from the bark and seed cones of *Magnolia officinalis *which has been used in traditional Chinese medicine. Recently, we have reported the chemopreventive effects of honokiol on UVB-induced skin cancer development in mice [17]. Honokiol and magnolol are isomers and share a number of biological properties. Studies have demonstrated that magnolol has multiple pharmacological properties such as antioxidant [18], anti-inflammatory [19], and central nervous system depressant effects [20]. It has been reported that magnolol delayed the formation of papillomas in mouse skin initiated by 7,12-dimethylbenz (α) anthracene (DMBA) and promoted by 12-*O*-tetradecanoyl phorbol-13-acetate (TPA) [21].

For the first time, in this study, we reported the effects of magnolol on UVB-induced skin cancer development in SKH-1 mice. Since UVB induces squamous cell carcinoma in mice, the effects of magnolol on human epidermoid squamous cell carcinoma A431 cells were investigated to elucidate the possible mechanisms of action. The effects of magnolol were investigated on UVB-induced skin carcinogenesis in SKH-1 mice, a model relevant to human cancer where UVB acts as complete carcinogen. Loss of apoptosis and rapid cell proliferation are major factors responsible for tumorigenesis [22], therefore, the present study focuses on the effects of magnolol on apoptosis, cell survival pathways and cell cycle arrest.

Signal transduction and activators of transcription 3 (STAT 3) and mitogen-activated protein kinase (MAPK) signaling play a major role in apoptosis, proliferation, and tumor promotion [23,24]. Overactivity of the MAPK pathway has been shown to be involved in cancer promotion and development [25-27]. Therefore, we investigated the effects of magnolol on the modulation of STAT3 and MAPK signaling pathways.

## Methods

### Reagents

Magnolol was purchased from Nacalai tesque (Kyoto, Japan). Thiazolyl blue tetrazolium bromide (MTT) and other chemicals of analytical grade were purchased from Sigma Chemical Co. (St. Louis, MO). Cell proliferation ELISA kit was purchased from Roche Diagnostics GmbH (Mannheim, Germany). Vibrant Apoptosis Kit 2 and APO-BrdU TUNEL assay kit were purchased from Molecular Probes (Eugene, OR). The primary antibodies such as cleaved caspase-3, cleaved caspase-8, pSTAT3-Tyr^705^, pSTAT3-Ser^445^, pMEK1/2, B-Raf and cleaved PARP were purchased from Cell Signaling Technology (Beverly, MA). Primary Antibodies such as Cdc25A, Cdc25B, Cdc25C, p-Cdc25C, CDK-2, CDK-4, Cyclin B1, Cyclin A, Cdc2p34, p-ERK1/2, p-AKT, PCNA, anti-mouse IgG horseradish peroxidase-linked and anti-rabbit IgG horseradish peroxidase-linked secondary antibodies were purchased from Santa Cruz Biotechnology (Santa Cruz, CA). Anti-Kip1/p27 antibody was purchased from BD-Pharmingen (San Diego, CA) and anti-Cip1/p21 antibody from Upstate Biotechnology (Lake Placid, NY).

### Cell culture

Human epidermoid carcinoma A431 cells were purchased from American Type Culture Collection (Manassas, VA). A431 cells were cultured in DMEM supplemented with 10% heat inactivated fetal bovine serum, 100 unit/ml of penicillin and 100 ug/ml of streptomycin in a humidified atmosphere containing 95% air and 5% CO_2 _at 37°C. For treatments of cell cultures, magnolol was dissolved in DMSO to make a 50 mM stock solution, this stock solution was diluted in DMEM at different concentrations and was immediately used. In all assays the final concentration of DMSO in DMEM was 0.4%.

### Animals

Five to six week old female SKH-1 mice were purchased from Charles River Laboratories (Wilmington, MA, USA). Institutional Animal Care and Use Committee (IACUC) approvals were obtained for all experimental protocols. Mice were housed under climate-controlled environment with a 12 hours light/dark cycle and were provided with free access to food and water during the experiment.

### UVB Light Source

Four FS-40-T-12 UVB lamps were used as UVB light source. UVB exposure dose was controlled by integrating dosimeters manufactured by Daavlin Corporation (Bryan, OH, USA).

### UVB-induced skin tumor development protocol

Five to six weeks old female SKH-1 mice were randomly divided into four groups of 20 each. Carcinogenesis was initiated and promoted by UVB, dose (30 mJ/cm^2^) for 5 days a week (monday-friday). This is a dose close to the UVB human exposure causing cancer development [28]. Group 1 served as control and received 200 μ l of acetone, group 2, group 3 and group 4 received 30 μ g, 45 μ g and 60 μ g of magnolol in 200 μ l of acetone respectively. Treatments were administered topically one hour before UVB exposure. The experiment was carried out for 25 weeks. Tumor counts and body weights were recorded on weekly basis for 25 weeks. Results were analyzed for tumor incidence, multiplicity and area.

### Histopathological analysis of mice tumors

Mice were euthanized by cervical dislocation at the end of the above mentioned protocol. Skin collected from five animals per group was fixed by immersion in 10% neutral buffered formalin for three days at room temperature. Fixed tissues were processed into paraffin-wax blocks, sectioned and stained with hematoxylin-eosin (HE) and then evaluated under light microscope.

### Effects of magnolol on tumor area in SKH-1 mice

Tumor areas were quantified as described earlier [17,29] by using images from tumor bearing mice which were taken at the end of 25 weeks. By using Photoshop CS3 (Adobe systems, San Jose, CA, USA) tumor boundaries were determined and areas were measured by using Image-Pro Plus 5.1 (Media Cybernetics, Inc, Bethesda, MD, USA).

### MTT assay for cell viability

A431 cells (9000 cells/well) were plated in 96 well plates. After 24 h, cells were treated with different concentrations of magnolol (75, 100, 125 μM) for 12 h, 24 h and 48 h, using cells treated with growth medium 0.4% DMSO as control. Cell viability was determined at the end of each treatment by using MTT assay as previously reported [30].

### BrdU assay for cell proliferation

A431 cells (9000 cells/well) were plated in 96 well plates. After 24 h, cells were treated with different concentrations of magnolol (75, 100, 125 μM) or treated with growth medium 0.4% DMSO as control, for 48 h. At the end of the treatment, Bromodeoxyuridine (BrdU) incorporation assay was carried out using ELISA kit (Roche Diagnostics, GmbH, Manheim, Germany) using manufacturer protocol as previously reported from our laboratory [30]. The experiment was repeated three times.

### Quantification of apoptosis by Annexin V/PI staining

Apoptosis was quantified by using Vibrant Apoptosis Kit 2 (Molecular Probes) as previously reported from our laboratory [30]. A431 cells (2 × 10^5^)/well were plated in six well plates and after 24 h were treated with magnolol (100, 150 μM) for 48 h. Then cells were collected, washed and stained with annexin-V labeled with a fluorophore that binds to phosphatidylserine exposed on apoptotic cells. Also cells were treated with propidium iodide (PI) a DNA intercalator dye that stains dead cells. Samples were analyzed after staining with both dyes in BD FACScan flow cytometry and the percentages of apoptotic cells were evaluated using CellQuest software (BD Biosciences, San Jose, CA).

### Quantitation of DNA fragmentation by TUNEL assay

Apo-BrdU TUNEL assay kit (Molecular Probes) was used to quantify the amount of DNA fragmentation in magnolol treated A431 cells by using manufacturer's protocol as previously reported [30]. Positive and negative control cells were run with each assay.

### Cell Cycle analysis

Subconfluent A431 cells plated in six well plates were treated with different concentrations of magnolol (75, 100, 125 μM) or control media for 12, 24 and 48 h. After each treatment, cells were harvested, washed and fixed in 70% ethanol in DPBS. Fixed cells were treated with 100 μ l of RNase A (1mg/ml) for 30 min at 37°C. After incubation, 900 μ l of staining buffer and 20 μ l of PI (Propidium iodide 1 mg/ml) were added to each sample and incubated for 30 min in the dark. The samples were analyzed with BD FACScan flow cytometry (BD Biosciences, San Jose, CA) using Cell Quest Software (BD Biosciences, San Jose, CA) as previously reported [31,32].

### Preparation of tissues and cell lysates for immunoblotting

Tissue samples: Mice were sacrificed by cervical dislocation, then treated skin was collected, fat from skin was removed by scalpel, and then skin was homogenized in 0.1 mM Tris-HCl/0.15 M NaCl (pH 7.4). The homogenate was filtered and centrifuged at 10000g for 45 min in a Beckman J2-21 centrifuge (Brea, CA.), the obtained pellet was combined with 5% SDS, 0.5% leupeptin and pepstatin and 1% PMSF, then was passed through a 25G needle and centrifuged at 13000g for 20 min, the obtained supernatant was heated for 5 min at 100°C. Finally, protein concentrations were determined by BCA protein assay (Pierce, Rockford, IL), then separated by SDS-PAGE and analyzed by Western blot.

For A431 cell lysates, 1.5 × 10^6 ^cells were plated in 100 mm culture dish. Subconfluent A431 cells were treated with varying concentrations of magnolol (75, 100, 125 μM) and DMEM 0.4% DMSO as control, for 24 and 48h. At the end of each treatment cells were lysed.

Protein concentrations for tissues and cells' proteins were determined by BCA protein assay kit (Pierce, Rockford, IL) with albumin as standard.

The tissues or cells' proteins (50 μ g) were resolved by SDS-PAGE and were transferred onto nitrocellulose membranes. The membranes were probed with appropriate antibodies followed by secondary antibody and detection by ECL plus detection system (Amersham Biosciences, Piscataway, NJ). Equal protein loading is ensured by reprobing each membrane with β-actin antibody. Western blotting was repeated for 3-5 samples and representative bands from all replicated experiments are reported. Western blots were detected and quantified by using a UVB Biochem Gel Documentation system (UVP, Inc., Upland, CA) and this data were analyzed statistically.

### Statistical Analysis

INSTAT software (Graph Pad, San Diego, CA) was used to analyze data. Chi square analysis was used for the data on tumor incidence. Analysis of variance followed by Tukey's test was used for tumor multiplicity and area, as well as for Western blots and for various *in vitro *assays. Significance in all experiments was considered at *p *< 0.05. All values were expressed as mean ± standard error.

## Results

### Effects of magnolol on weight gain and skin appearance

Pretreatment of animals with magnolol at all doses did not have any effects on weight gain (data not shown) and skin appearance of mice indicating the safety of magnolol at these doses.

### Chemopreventive effects of magnolol on UVB-induced skin tumorigenesis

**Figure 1:**
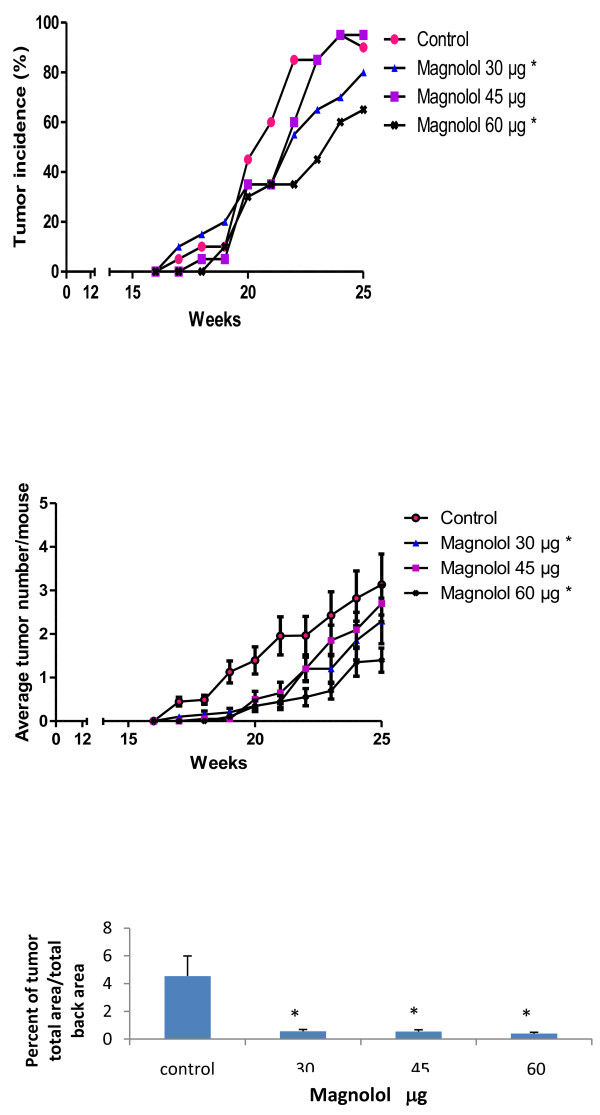
**Effects of magnolol pretreatment on tumor incidence, tumor multiplicity and tumor area in UVB-induced skin carcinogenesis in SKH-1 mice**. **(A) **Effects of topical magnolol pretreatment on tumor incidence. From the 20^th ^week to the end of the experiment magnolol 30 μ g and 60 μ g reduced significantly tumor incidence. Each point represents the percentage of animals bearing at least one tumor, values derived from 20 mice. *Significant difference (*p *< 0.05). **(B) **Effects of magnolol pretreatment on tumor multiplicity. Magnolol 30 and 60 μ g pretreatment significantly decreased tumor multiplicities from the 16^th ^to 25^th ^week of UVB induced carcinogenesis. Each point represents mean number of tumors per mouse ± SE derived from 20 mice. *Significant difference (*p *< 0.05). **(C) **Effects of magnolol treatment on tumor area. Average ratio of total tumor area to total back area of the SKH-1 mice. Each bar represents mean ratio of tumor area per mouse ± SE derived from 20 mice.* Significant difference (*p *< 0.05)

The effects of magnolol pretreatment on the tumor incidence in SKH-1 mice are shown in Figure 1A. Tumor incidence was 100% in both the control and magnolol pretreated group (45 μ g) by the end of 25 weeks. Magnolol pretreatments with 30 and 60 μ g per application delayed the appearance of tumors as compared to control and 45 μ g magnolol applications. The results showed that tumor incidence was significantly lower during 21-25 weeks (*p *< 0.05) in the magnolol pretreated groups (30 and 60 μ g) as compared to control group. Overall, the magnolol pretreatments (30, 60 μ g) decreased tumor incidence compared with control at the end of the experiment. Interestingly, 45 μ g application of magnolol did not have any significant effect on UVB-induced tumor incidence.

The effects of magnolol pretreatment on tumor multiplicity are shown in Figure 1B. Topical application of 30, 45, 60 μ g of magnolol prior to UVB treatments showed protection against skin tumor development in SKH-1 mice. We found that tumor multiplicity is significantly (*p *< 0.05) decreased in the magnolol pretreated groups (30, 60 μ g) from 16 weeks to 25 weeks when compared to control group. At the end of the experiment, magnolol 30 μ g and 60 μ g pretreatments resulted in 27-55% decrease in tumor multiplicity respectively. Interestingly, the 45 μ g application of magnolol had lesser effects than the 30 μ g application, similar to the results for tumor incidence.

**Figure 2:**
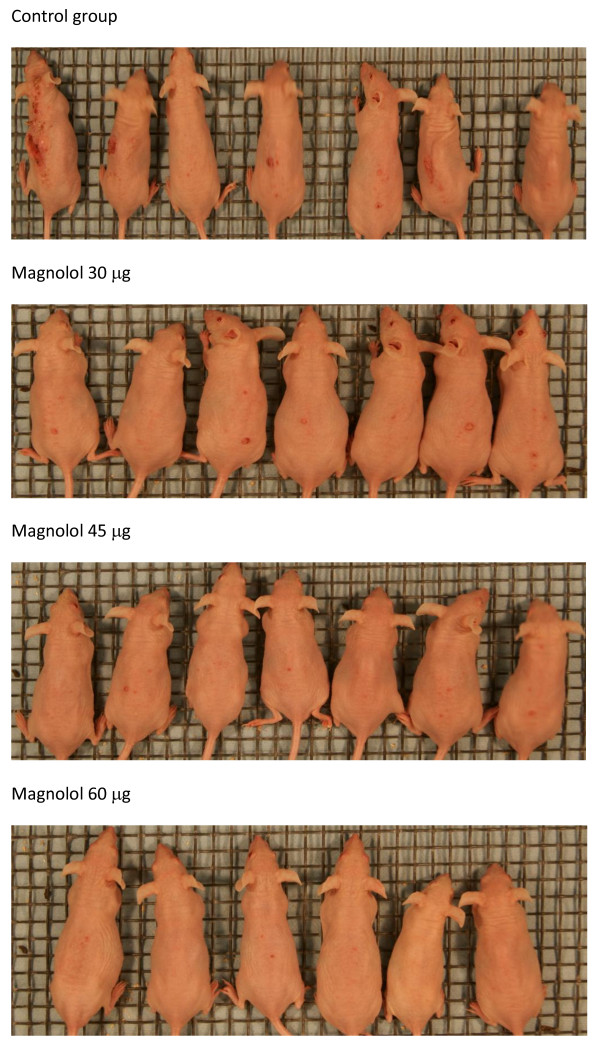
**Effects of magnolol treatment on UVB induced skin tumors in SKH-1 mice**. Mice were exposed five days a week to 30 mJ/cm^2 ^UVB one hour before different topical treatments. Control group (n = 20) was treated with 200 μ l of acetone. Treatment groups (n = 20 each) received 30, 45 or 60 μ g of magnolol dissolved in 200 μ l of acetone. Pictures were taken at the end of the 25^th ^week. Animals were randomly chosen for the pictures.

The effects of magnolol pretreatment on the ratio of total tumor area to total back area are shown in Figure 1C. In the control and magnolol pretreated groups (30, 45 and 60 μ g) the mean ratio of tumor area to total back area was 4.5%, 0.5%, 0.5%, 0.3% respectively, resulting in 87 - 93% reduction in tumor area with magnolol pretreatments compared to control. Unlike the data on tumor incidence and multiplicity, effects of 45 μ g application of magnolol had similar effects as 30 and 60 μ g applications. A representative picture showing gross appearance of the animals is shown in Figure 2.

The histopathological examination of the tumors after 25 weeks of treatments indicated that control and magnolol treated groups developed squamous cell carcinoma in the skin (Pictures not shown).

### Effects of magnolol on apoptotic proteins in SKH-1 mice

**Figure 3:**
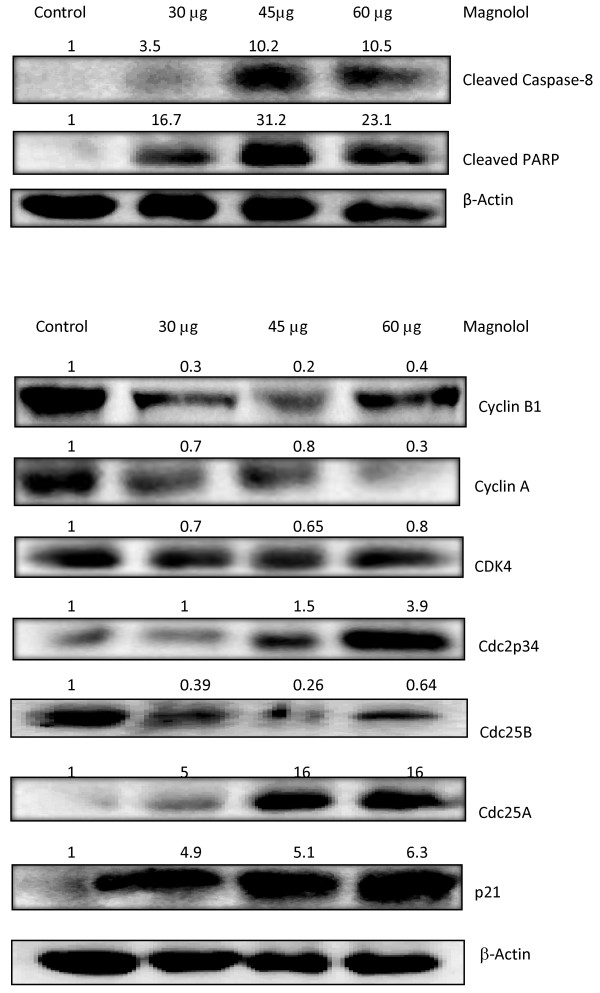
**Effects of magnolol on the expression and activation of proteins in UVB-induced photocarcinogenesis in SKH-1 mice**. **(A) **Effects of magnolol on the cleavage of apoptotic proteins. Proteins were isolated from epidermal tissues of mice, lysates were prepared and subjected to Western blot analysis. β-actin was used to verify equal loading of the samples for each membrane. Bands are representative from three experiments. **(B) **Effects of magnolol pretreatment on the expression of cell cycle proteins. Proteins were extracted from the mice's back skin as mentioned in materials and methods. Lysates were subjected to Western blot analysis. β-actin was used as loading control. Bands are representative from three experiments.

Epidermal lysates from mice skin of both control and magnolol pretreated groups were prepared at the end of the study. The effects of magnolol on caspase-8 and PARP cleavage, key proteins in apoptosis, are shown in Figure 3A. Topical application of magnolol significantly (*p *< 0.05) increased the cleavage of caspase-8 and PARP as compared to control.

### Effects of magnolol on cell cycle proteins in SKH-1 mice

Our studies on the effects of magnolol on human epidermoid carcinoma A431 cells indicated that magnolol caused cell cycle arrest at G2/M phase (results reported later in the manuscript). Therefore, we investigated various proteins involved in G2/M phase of the cell cycle in skin samples collected from the various experimental groups. The effects of magnolol on cell cycle proteins from skin of experimental mice are shown in Figure 3B. Pretreatment of magnolol decreased the expression of Cyclin B1, Cyclin A, CDK-4 and Cdc25B but increased expression of Cdc2 and Cdc25A as compared to control. Topical application of magnolol to SKH-1 mice resulted in increased expression of the cell cycle inhibitor p21.

In order to further elucidate the mechanism of action of magnolol, *in vitro *effects of various concentrations of magnolol on human epidermoid carcinoma A431 cells were investigated.

### Magnolol treatment decreased cell viability in A431 cells

**Figure 4:**
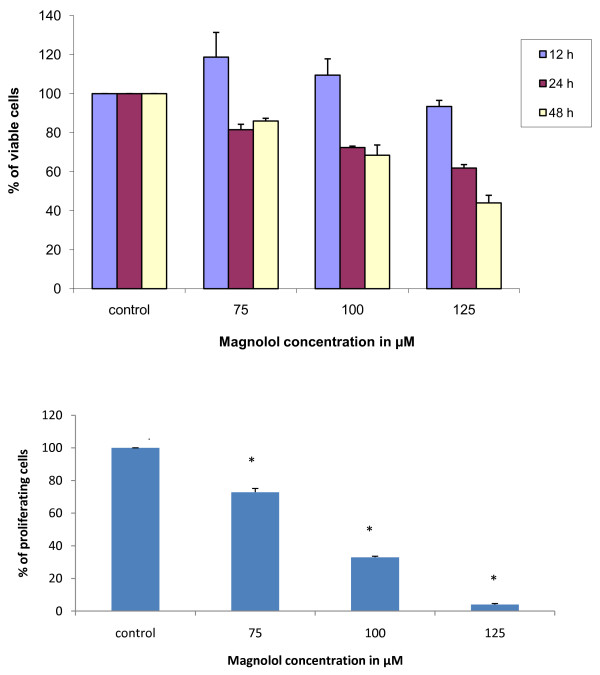
**Effects of magnolol on cell viability and cell proliferation in A431 cells**. **(A) **Effects of magnolol on cell viability. A431 cells were treated with control media or magnolol (75-125 μM) for 12, 24 and 48 hours. At the end of the respective treatment MTT assay was performed. Values are expressed as mean ± SE of eight replicates in each treatment. **(B) **Effects of magnolol on cell proliferation in A431 cells. Cells were treated in similar conditions as described for MTT assay for 48 hours, and then BrdU incorporation assay was performed. Values are expressed as mean ± SE of three replicates in each treatment.

As this is the first time the effects of magnolol on human epidermoid carcinoma A431 cells are investigated, MTT assay was conducted to determine the effects of magnolol on cell viability. A431 cells double in 24 hours [33,34]; therefore we studied the effects of magnolol treatment at 12, 24 and 48 hours. Magnolol treatment (75-125 μM) showed a concentration and time dependent decrease in cell viability Figure 4A. Magnolol treatment did not show a significant effect at 12 h, but at 24h and 48 h treatment significantly decreased cell viability. The effects of magnolol at 48h may not be due to cell death but due to proliferation inhibition. As shown in Figure 4A, cell viability of magnolol treated cells compared to controls ranged from 100-98% at 12h, 80-70% at 24 h and 80 - 50% at 48 h.

### Magnolol inhibited cell proliferation in A431cells

We investigated the effects of magnolol on cell proliferation in A431cells by BrdU incorporation assay. Magnolol (75-125 μM) at 48 hours treatment resulted in a 30 - 96% decrease in cell proliferation as compared to control. Figure 4B.

### Magnolol induces apoptosis in A431 cells

**Figure 5:**
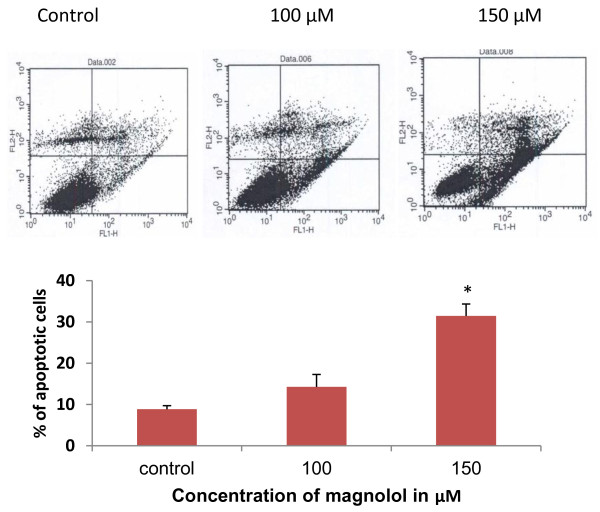
**Effects of magnolol on apoptosis in A431 cells as assessed by annexin-V/PI staining**. Cells were treated with magnolol (0-150 μM) for 48 h, at the end of the treatment adherent and non-adherent cells were collected and treated with annexin-V labeled with a fluorophore, which can identify apoptotic cells by binding to phosphatidylserine exposed on apoptotic cells; and with propidium iodide that stained dead cells. Dot plot of annexin-V (FL1-H)/PI (FL2-H) staining of A431 cells by flow cytometry. The lower right quadrant shows early apoptotic cells that are labeled with annexin-V, having green fluorescence. The upper right quadrant stained by annexin-V and PI indicates late apoptotic cells. The lower left quadrant contains viable cells which exclude PI and are negative for annexin-V staining, and the upper left quadrant are necrotic cells stained by PI only. The bar graph describes the percentages of apoptotic cells after each treatment. In each case data represent mean ± SE of three observations. *p < 0.05 indicates statistical significant difference in magnolol treated groups compared with the control.

To investigate whether cell death caused by magnolol is an apoptotic response, cells were treated with magnolol (100, 150 μM) for 48 h, followed by annexin-V/PI staining using a Vibrant Apoptosis kit. The stained cells were analyzed through flow cytometry. As shown in Figure 5, early apoptotic cells are represented in the lower right quadrant and late apoptotic cells in the upper right quadrant. The results showed that magnolol treatments (100 and 150 μM) resulted in 14.2% and 31.4% of apoptosis respectively compared with DMEM 0.4% DMSO treated control showing 8.8% of apoptotic cells. These results suggest magnolol treatment induced a significant degree of apoptosis (*p < 0.05*) in a concentration dependent manner. This data supports the results from our animal experiments.

### Magnolol induces DNA fragmentation in A431 cells

**Figure 6:**
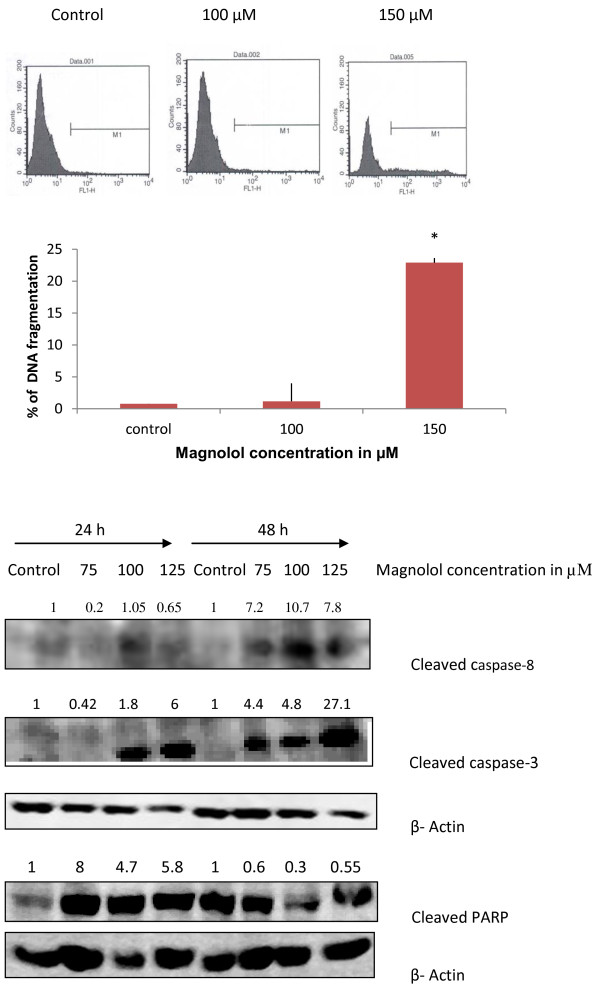
**Effects of magnolol on DNA fragmentation and activation of apoptotic proteins in A431 cells**. **(A) **DNA fragmentation. Cells were treated with magnolol (0-150 μM) for 48 hours, at the end of the treatment, adherent and non-adherent cells were collected and subjected to TUNEL assay. The gate M1 includes the apoptotic cells with fragmented DNA, which were positive for green fluorescence. The bar graph indicates the percentages of apoptotic cells with fragmented DNA. In each case, data represents mean ± SE of three observations. *p < 0.05 indicates statistical significant difference in magnolol treated groups compared with the control. **(B) **Effects of magnolol on the activation of apoptotic proteins in A431 cells. Cells were treated with magnolol for 24 and 48 h, cells were then collected. Cell lysates were prepared and subjected to SDS-PAGE and Western blot analysis. Membranes were probed with appropriate antibodies. Pictures are representative from three experiments.

TUNEL assay was performed in order to investigate the effects of magnolol on DNA fragmentation, which is hallmark of late apoptosis that commits cells to die. As shown in Figure 6A, M1 gate is used to indicate DNA fragmented cells. Compared with the DMEM 0.4% DMSO treated control showing 0.8% of DNA fragmentation, magnolol treated A431 cells at 100 and 150 μM resulted in 1.17% and 21% of DNA fragmentation after 48 hours treatment. These results suggest that 100 μM did not induce DNA fragmentation whereas 150 μM concentration significantly increased DNA fragmentation. Figure 6A.

### Magnolol induces cleavage of caspases and PARP during apoptosis in A431 cells

Western blot analysis for caspases was used to further investigate magnolol induced apoptosis in A431 cells. The results showed that magnolol treatment increased the expression of cleaved caspase-8, and cleaved caspase-3 in a concentration dependent manner. We observed increased cleavage of PARP only at 24 h, membranes were checked for equal protein loading using β-actin as control. Figure 6B.

### Magnolol induces G2/M cell cycle arrest

**Figure 7:**
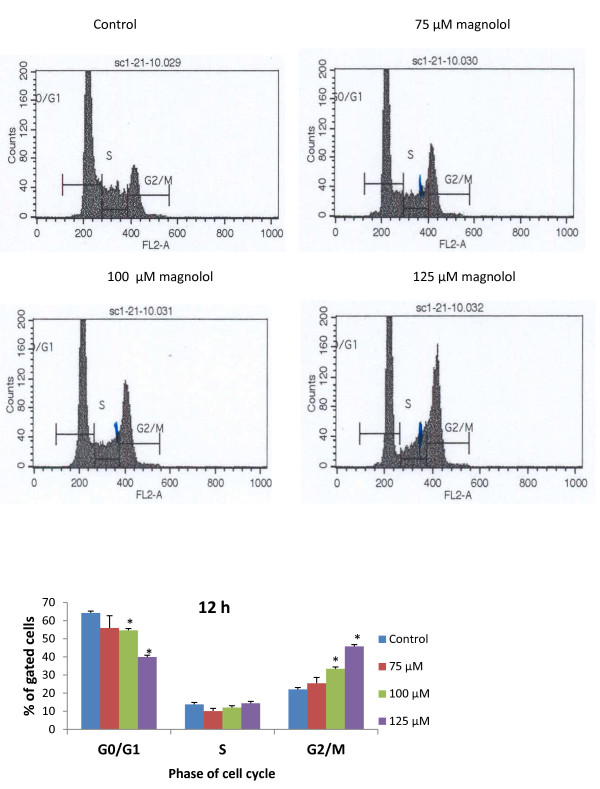
**Effects of magnolol on the cell cycle phases distribution in A431 cells**. **(A) **Cell cycle histograms. Cells were treated with magnolol (0, 75, 100 and 125 μM) for 12 h. At the end of the treatment, cells were harvested and digested with RNase. Cellular DNA was stained with propidium iodide and analyzed by flow cytometer as described in the Materials and Methods. (**B) **Data from the cell cycle distribution histograms were summarized into a bar graph and presented as the mean ± SE of three observations. *p < 0.05 indicates statistical significance in magnolol treated groups as compared to the control.

To determine the mechanism involved in antiproliferative activity, the effects of magnolol on cell cycle progression were studied in A431 cells. The effects of magnolol on the cell cycle were determined following treatment with 75, 100 and 125 μM of magnolol for 12 h, 24 h, and 48 h. As shown in Figure 7A and 7B, magnolol treatment resulted in a significantly increased number of cells in G2/M phase following 12 h at 100 μM (33.45%) and 125 μM (45.79%) compared with the control (22%). The concentration dependent effect of magnolol on G2/M arrest is at the expense of the G0/G1 phase (Figure 7B). These results are in agreement with the data from animal experiments (Figure 3B).

### Magnolol decreases expressions of G2/M regulatory proteins Cdks and cyclins and increased Cip1/p21 in A431 cells

**Figure 8:**
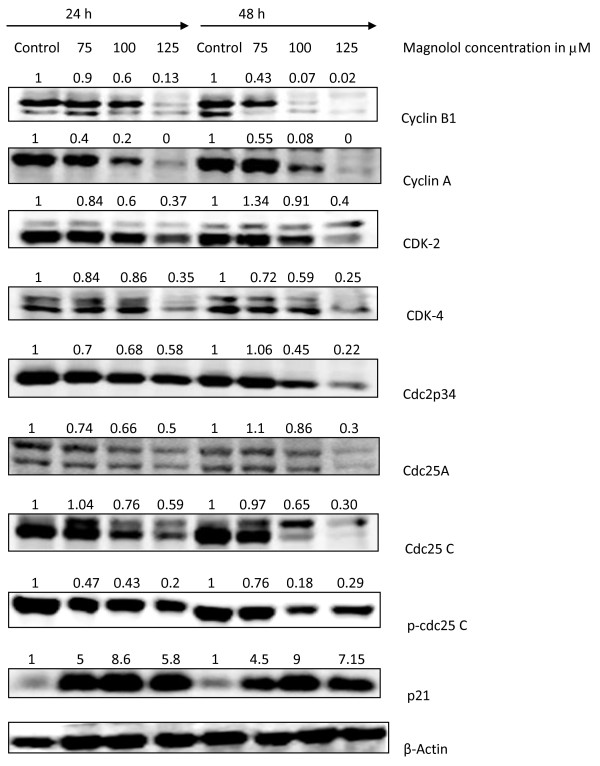
**Effects of magnolol on the expression of cell cycle regulatory proteins in A431cells**. Cells were treated with varying concentrations of magnolol (0, 75, 100, 125 μM) for 24 and 48 h and thereafter cell lysates were prepared. Total cell lysates were subjected to SDS-PAGE followed by Western blot analysis. β-actin was used to verify equal loading of samples

As cell cycle progression is dependent on various cyclins and cyclin-dependent kinases (CDK's), we focused our interest on investigating the expression of A431cell cycle proteins after magnolol treatment. Magnolol treatment resulted in strong inhibition in the expression of cyclin B1 (a protein involved in M phase) and cyclin A (a protein involved in both S and G2 phases) in a concentration and time dependent manner with almost disappearance of bands with higher concentrations. Magnolol treatment also decreased the expression of CDK2 and CDK4 in a concentration dependent manner at 24 h and 48 h. Reduction of CDK4 is more pronounced than CDK2 (Figure 8)

To further elucidate the mechanisms involved in the G2/M cell cycle arrest after magnolol treatment, we investigated various proteins involved in the G2/M phase. Magnolol treatment to A431 cells resulted in a decreased expression of Cdc2p34, Cdc25A, Cdc25C and pCdc25C (Ser216). All these results taken together suggest that magnolol induces G2/M cell cycle arrest through the modulation of G2/M regulatory proteins (Figure 8).

We next assessed the effects of magnolol on the expression of Cip1/p21, a cyclin dependent kinase inhibitor which is known to regulate the cells at the G1-S check point [35]. Magnolol treatment to A431 cells resulted in a significant increase in the expression of p21 in a concentration dependent manner compared with control cells. Collectively all these results suggest that increase in CDK inhibitory protein p21 by magnolol may have a role in cell cycle arrest in G2/M phase of A431 cells (Figure 8).

### Magnolol inhibits STAT3 phosphorylation in A431 cells

**Figure 9:**
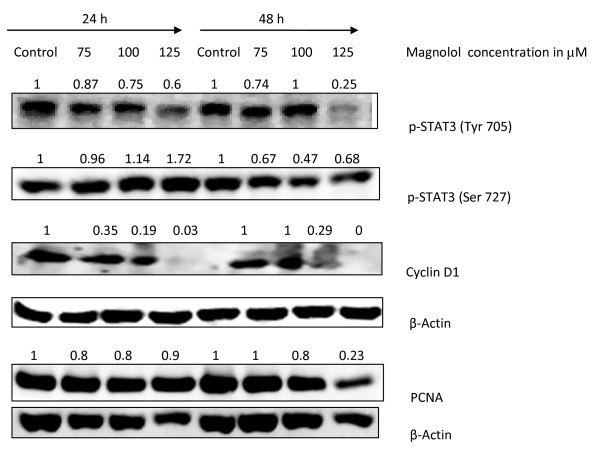
**Effects of magnolol on STAT3 phosphorylation in A431 cells**. Sub confluent cells were treated with 0, 75, 100 and 125 μM of magnolol for 24 and 48 h. At the end of each treatment, cells were harvested and total cell lysates were evaluated by Western blotting for phosphorylation of STAT3 (Tyr 705 and Ser 727), PCNA and cyclin D1. Protein loading was verified by reprobing membrane for β-Actin.

In order to investigate the molecular mechanism of magnolol in A431 cells, we first assessed the effects of magnolol on STAT3 phosphorylation. The effects of magnolol on STAT3 phosphorylation are shown in Figure 9. Compared with control treated cells, magnolol treated cells showed inhibition of STAT3 phosphorylation at Tyr705 at 24 and 48 h, as well as inhibition of phosphorylation of STAT3 at Ser 727 for 100 and 125 μM at 48 h. Magnolol treatment resulted in a time and concentration dependent decrease in p-STAT3 Tyr705. Downstream targets of STAT3 include PCNA and cyclin D1 [24]. We found that magnolol treatment decreased the expression of these proteins (Figure 9).

### Effects of magnolol on B-Raf, p-MEK, ERK and AKT in A431 cells

**Figure 10:**
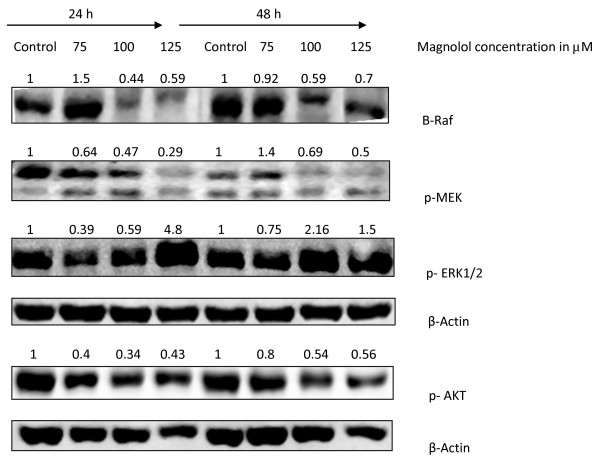
**Effects of magnolol on B-Raf expression and MEK/ERK/AKT phosphorylation in A431 cells**. Cells were treated with varying concentrations of magnolol (0-125 μM) for 24 and 48 h. Cells were harvested and total cell lysates were prepared at the end of each treatment and subjected to Western blot analysis for various proteins.

We next assessed the effects of magnolol on proliferation markers. MAPK signaling pathway play an important role in cell proliferation, and cell growth arrest [36]. We investigated the effects of magnolol on B-Raf, p-MEK, p-ERK in A431 cells at 24 and 48 h. Results showed that magnolol treatment decreased the expression levels of B-Raf and phosphorylation of MEK in a concentration dependent manner (Figure 10). Our results showed that ERK activation is increased for 125 μM at 24 and 48 h suggesting that magnolol induces cell growth inhibition by activating ERK. In addition to this, we found that magnolol treatment decreased the phosphorylation of AKT.

## Discussion

Magnolol, a hydroxylated biphenolic compound isolated from *Magnolia officinalis*, most commonly used in traditional Chinese medicine has been investigated for its effects on skin carcinogenesis. In this study, we determined the effect of magnolol in UVB-induced skin cancer in SKH-1 mice and on a human epidermoid skin cancer cell line *in vitro*. Neolignans from *Magnolia officinalis *delayed papilloma formation in skin tumor promotion by TPA [21,37]. We investigated the effects of magnolol in a UVB-induced skin carcinogenesis model with a UVB dose of 30 mJ/cm^2^/day which is more translational and relevant to human skin cancer as compared to previous studies that used higher doses of UV radiation [5,15]. Magnolol 30 μg and 60 μg in 200 μl of acetone showed a protective effect in a dose dependent manner when applied topically. In this study interestingly, 45 μg magnolol did not have any effect on tumor incidence and lower effects than the 30 μg application in tumor multiplicity. Magnolol may have biphasic effects on various target proteins not investigated in this study, thus the middle dose is less effective than the lower dose. Further studies with an increased range of magnolol doses are needed to fully understand this biphasic effect. We used very low doses (in micrograms) of magnolol compared to other chemopreventive agents which use milligrams per applications [5,13] thus indicating the higher potency of magnolol over other agents. The results demonstrated that magnolol delayed the onset of tumorigenesis when compared to the control. Tumor multiplicity was reduced by 27-55% for 30 μg and 60 μg of magnolol respectively compared to the control.

Mechanistic studies showed that magnolol induced apoptosis through extrinsic pathway and affected tumor development by causing cell cycle arrest at G2/M phase in our animal models.

To gain insight and have understanding of signaling mechanisms involved in the magnolol anticarcinogenic effect, we used human epidermoid A431 cells as an *in vitro *model. Magnolol inhibits cell viability and proliferation which together contributed to overall inhibition of cell growth in A431 cells at concentrations 75-125 μM for 12-48 h. Cancer development involves deregulation in cell cycle progression. Control of the cell cycle plays an important role in controlling tumor growth [22,38]. As such, effects of magnolol on the cell cycle and its related proteins were investigated in A431 cells. The results obtained demonstrate that magnolol induced G2/M cell cycle arrest, is one mechanism of inhibition of cell viability and proliferation. As cyclins/cyclin dependent kinases tightly regulate the cell cycle progression [39,40], the effects of magnolol on cell cycle proteins were investigated. Our findings revealed that treatment of cells with magnolol resulted in a significant decrease in cyclin A, cyclin B1, CDK2, CDK4, Cdc2 and increase in Cip1/p21 expression at all concentrations compared to control. Our studies on honokiol [17], an isomer of magnolol have indicated similar anticarcinogenic effects as magnolol. However, honokiol caused cell cycle arrest at G0/G1 phase in A431 cells [41] unlike magnolol which caused cell cycle arrest at G2/M phase.

Anticarcinogenic effects are modulated by two major events: inhibition of cell proliferation and induction of apoptosis [25,42]. Accordingly, the effects of magnolol on induction of apoptosis in A431 cells were investigated. During apoptosis, cells undergo changes such as loss of phospholipids asymmetry of the plasma membrane, cell shrinkage, proteases activation and finally DNA fragmentation [43]. Our flow cytometry data demonstrated that magnolol significantly induced apoptosis in A431 cells as assessed by annexin-V/PI staining which detects apoptotic cells by their loss of phospholipids plasma membrane asymmetry. Then, later we examined DNA fragmentation in apoptotic cell by using TUNEL assay. Magnolol 48h treatment induced DNA fragmentation in A431 cells at higher concentrations (150 μM).

There are two reported pathways for the induction of apoptosis. In the extrinsic or death receptor pathway of apoptosis, activation of death receptors by ligands leads to activation of caspase-8. This activated caspase-8 can activate caspase-3, an executioner caspase. Activated caspase-3 can cleave PARP and thereby results in apoptosis [44,45]. Consistent with the above reports, magnolol treatment to A431 cells activated caspase-8 and caspase-3 in a concentration dependent manner that led to PARP cleavage. These observations suggest that magnolol induced apoptosis through extrinsic pathway and are consistent with the results obtained from animal experiments.

The STAT pathway regulates the transcription of a wide variety of genes involved in proliferation, development, and tumorigenesis [46,47]. Among different STAT family members, STAT3 is implicated in tumorigenesis [47] and it plays an important role in skin cancer development [48]. STATs are activated either by serine or tyrosine phosphorylation by JAK kinases, then they undergo dimerization followed by nuclear translocation and regulation of the expression of target genes [49]. Our results showed that treatment of A431 cells with magnolol inhibited the phosphorylation of STAT3 at tyrosine residues. Downstream targets of p-STAT3 include cyclin D1, our results showed that magnolol decreased cyclin D1 expression, and this may lead to cell cycle arrest [50]. In the present study, our *in vitro *data has shown that magnolol treatment increased the phosphorylation of ERK protein in A431 cells, suggesting activation of ERK and upregulation of p21 by magnolol as a mechanism for cell cycle arrest [51]. However, further studies are needed to study the effects of magnolol on the phosphorylation of these proteins at very early stages instead of at 24h and 48h.

Magnolol inhibited cell proliferation through regulation of Cip1/p21 in human glioblastoma cells [52], induced apoptosis via inhibition of EGFR, PI3K/AKT signaling pathways in human prostate cancer cells [36] and inhibited MMP-9 expression through the transcription factor NF-kB in TNF-α- induced human urinary bladder cancer cells [53]. Magnolol induces apoptosis via activation of both mitochondrial and death receptor pathways in A375-S2 malignant melanoma cells [54]. Recent studies by Tanaka et al. [55], have shown the preventive effects of magnolol on UV-induced photoaging by inhibiting the expression of NF-kB. A recent study by Kuo et al. [35] showed that magnolol down-regulated TPA induced iNOS and COX-2 gene expression in mouse skin suggesting that magnolol could be novel agent preventing inflammation associated tumorigenesis.

Our studies for the first time provided the evidence that magnolol pretreatment at very low doses (micrograms per applications compared to most other agents which are used in milligrams per application) prevents UVB-induced skin cancer development in SKH-1 mice by both inducing apoptosis and decreasing cell proliferation through modulation of various signaling pathways. The sunscreen effects of magnolol have not been investigated in this study which may contribute to anticarcinogenic effects. Future studies involving various inhibitors, antisense oligonucleotides and dominant negative mutants or siRNA are needed to map the pathways to conclude the signaling involved in the anticancer effects of magnolol. Magnolol has a great potential to be a safe and potent chemopreventive agent against skin cancer development in human.

## Conclusions

Our studies for the first time provided the evidence that magnolol pretreatment at very low doses (micrograms per application compared to most other agents which are used in milligrams per application) prevents UVB-induced skin cancer development in SKH-1 mice both by inducing apoptosis and decreasing cell proliferation through modulation of various signaling pathways. Magnolol has a great potential to be a safe and potent chemopreventive agent against skin cancer development in human.

## Competing interests

The authors declare that they have no competing interests.

## Authors' contributions

CC has performed UVB induced skin cancer development in SKH-1mice, *in vitro *assays and Western blots for various proteins for A431cells and wrote the manuscript; RG performed Western blots for cell cycle and apoptotic proteins from tissue samples. XZ helped with Western blots. RSK and AY helped with flow cytometric analysis; DZ performed the histopathological examination of samples; MH helped with image pro analysis; HF identified the magnolol for the studies; and CD overall conceived and executed the idea, and prepared the final draft of manuscript. All authors read and approved the final manuscript.

## Pre-publication history

The pre-publication history for this paper can be accessed here:

http://www.biomedcentral.com/1471-2407/11/456/prepub
